# Analysis of allele-specific expression of seven candidate genes involved in lipid metabolism in pig skeletal muscle and fat tissues reveals allelic imbalance of *ACACA*, *LEP*, *SCD*, and *TNF*

**DOI:** 10.1007/s13353-019-00485-z

**Published:** 2019-01-26

**Authors:** Monika Stachowiak, Krzysztof Flisikowski

**Affiliations:** 1 Department of Genetics and Animal Breeding, Poznan University of Life Sciences, Wolynska 33, 60-637 Poznan, Poland; 2 Chair of Livestock Biotechnology, Technical University of Munich, Liesel-Beckmannstr. 1, 85354 Freising, Germany

**Keywords:** Adipose tissue, Allele-specific expression, CpG methylation, Fatness, Pig, Skeletal muscle

## Abstract

Analysis of allele-specific expression may help to elucidate the genetic architecture of complex traits including fat deposition in pigs. Here, we used pyrosequencing to investigate the allele proportions of candidate genes (*ACACA*, *ADIPOR1*, *FASN*, *LEP*, *ME1*, *SCD*, and *TNF*) involved in regulation of lipid metabolism in two fat deposits (subcutaneous and visceral fat) and *longissimus dorsi* muscle of pigs representing Polish Large White, Polish Landrace, Duroc, and Pietrain breeds. We detected differential allelic expression of *ACACA*, *LEP*, *SCD*, and *TNF* in all tissues analyzed. To search for putative *cis*-regulatory elements involved in allele-specific expression, we quantified the methylation level within CpG islands located in 5′-flanking regions of *ACACA* and *SCD*. Comparison between samples showing markedly disproportionate allelic expression and control groups with similar levels of both alleles did not reveal significant differences. We also assessed the association of rs321308225 (c.*195C>A) an SNP located in the 3′UTR of *ACACA* with its allelic expression in Polish Landrace pigs, but it was not significant. We conclude that allelic imbalance occurs frequently in regard to genes involved in regulation of lipid deposition in pigs, and further studies are necessary to identify *cis*-regulatory elements affecting *ACACA*, *LEP*, *SCD*, and *TNF* expression in porcine fat tissues and skeletal muscle.

**Electronic supplementary material:**

The online version of this article (10.1007/s13353-019-00485-z) contains supplementary material, which is available to authorized users.

## Introduction

Preferential expression of one particular allele rather than the other is a common phenomenon across tissues and species (Chamberlain et al. 2015; Gaur et al. 2013). Such allelic imbalance can be a consequence of functional DNA polymorphisms or epigenetic factors including DNA methylation, chromatin modifications, nuclear positioning, or noncoding RNAs that affect gene expression in a *cis*-acting manner (Font-Cunill et al. 2018; Gaur et al. 2013; Takizawa et al. 2008). Analysis of allele-specific expression may be helpful in revealing the genetic basis of complex traits such as predisposition to obesity in humans and immunity traits in pigs (Knowles et al. 2017; Maroilley et al. 2017)*.*

The modern pig industry is focused on efficient production and high meat quality. Adipose tissue accumulation is an important production trait in pigs that is affected by a combination of environmental and genetic factors, including the variation of gene expression in adipocytes and skeletal muscles (Stachowiak et al. 2016; Switonski et al. 2010). The analysis of allelic imbalance may facilitate the elucidation of genetic or epigenetic determinants of porcine fatness and improve the understanding of human obesity.

Here, we investigated the allelic expression of seven candidate genes involved in the regulation of lipid metabolism in fat deposits and skeletal muscle sampled from commercial pig breeds and attempted to decipher the effects of putative *cis*-regulatory elements on allelic imbalance of *ACACA* and *SCD*.

## Materials and methods

One hundred forty-two gilts representing Polish Large White (PLW; *n* = 48), Polish Landrace (PL; *n* = 35), Duroc (*n* = 38), and Pietrain (*n* = 21) breeds were reared under similar environmental conditions, fed ad libitum with the same commercial mix fodder, slaughtered at 100–105-kg weight, and dissected at the Pig Testing Station in Pawlowice (Poland). Peripheral blood, *longissimus dorsi* (*l. dorsi*) muscle, subcutaneous, and visceral fat tissues were collected.

Genomic DNA (gDNA) was extracted from blood, and exonic reporter SNPs (rSNPs) in *ACACA* (*acetyl-CoA carboxylase alpha*): rs81303284 (c.*99A>T); *ADIPOR1* (*adiponectin receptor 1*): rs81508987 (c.759G>A); *FASN* (*fatty acid synthase*): rs324640280 (c.339C>T); *LEP* (*leptin*): rs45431504 (c.289T>C); *ME1* (*malic enzyme 1*): rs328566530 (c.582T>C); *SCD* (*stearoyl-CoA desaturase*): rs334462984 (c.*931C>G); and *TNF* (*tumor necrosis factor*): rs80945725 (c.306A>G) were genotyped by capillary sequencing on a 3130 Genetic Analyzer (Applied Biosystems).

RNA was extracted from subcutaneous, visceral fat and *l. dorsi* muscle. Prior to reverse transcription, RNA samples were digested with DNase I to remove contaminating gDNA. Allele quantification assays were designed with the use of PyroMark Assay Design 2.0 software (Qiagen). All primers obtained high-quality scores and only primers annealing to a DNA template without mutation sites were used. Allele proportions for each rSNP in cDNA and gDNA were measured by pyrosequencing of cDNA and gDNA using Pyromark Q48 Autoprep system (Qiagen). Allelic ratios were calculated by dividing the percentage of one allele by the other. Any bias resulting from variations in nucleotide incorporation during pyrosequencing reaction was normalized for each gene by dividing the allelic ratio of cDNA and gDNA samples by the mean allelic ratio from gDNA. The bi-directional character of ASE was neutralized by dividing the higher percentage by the lower, as described by Olbromski et al. (2013). Next, the allelic transcript ratios were log_10_-transformed and the mean allelic expression between cDNA and gDNA for each breed and tissue was compared by two-tailed *t* test with unequal variances (Forton et al. 2007; Murani et al. 2009). The methylation status was determined for CpG islands (CGi) localized in 5′-flanking regions of *ACACA* (chromosome 12; CGi1: 38,874,595…38,875,705 and CGi2: 38,824,323…38,825,369) and *SCD* (chromosome 14: 111,460,649…111,461,395) based on porcine genome data (Sscrofa11.1, NC_010454.4). Genomic DNA was isolated from visceral fat and skeletal muscle and bisulfite-converted. 5-Methylcytosine (5-mC) levels (%) were quantified using Pyromark Q48 Autoprep pyrosequencer (Qiagen) and compared with Mann-Whitney Rank Sum Test between samples showing similar (*n* = 10 for *ACACA* in visceral fat; *n* = 10/9 for *SCD* in visceral fat/*l. dorsi* muscle) and extremely imbalanced allelic expression (*n* = 7 for *ACACA* in visceral fat; *n* = 5/9 for *SCD* in visceral fat/*l. dorsi* muscle).

Candidate SNPs in *ACACA*: rs321308225 (c.*195C>A) and *TNF*: rs328373700 (c.-791C>T) were genotyped using capillary sequencing. Due to the genotype distribution, association between analyzed SNP and allelic transcript expression was performed for rs321308225 in the PL breed only, using a two-tailed *t* test with unequal variances for log_10_-transformed allelic transcript ratios of homozygous versus heterozygous samples, as described by Murani et al. (2009).

All primers used in the study are listed in Suppl. Table S1. For a detailed description of methods, please see Stachowiak et al. (2018).

## Results and discussion

**Fig. 1:**
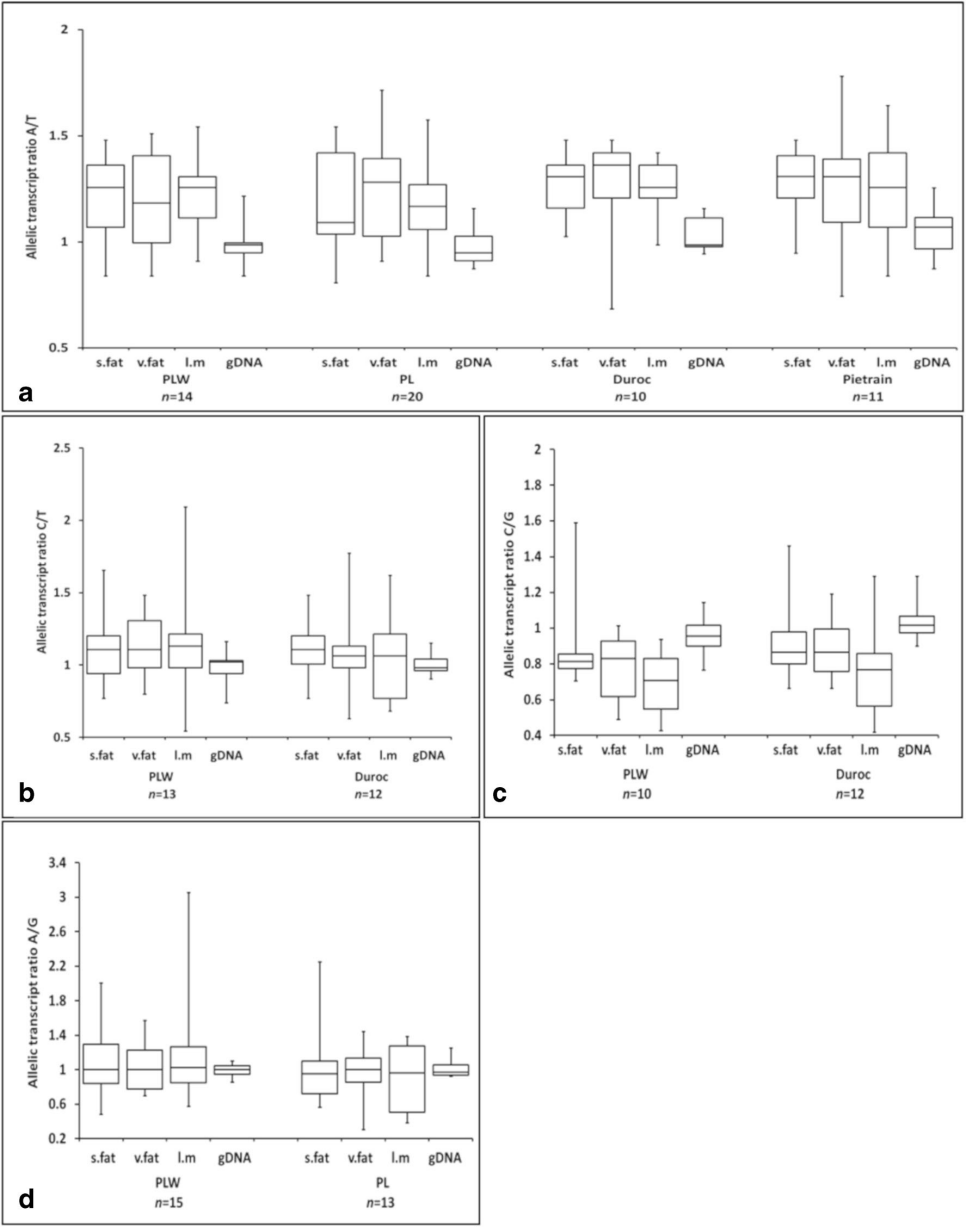
Distribution of allelic ratios for **a***ACACA*, **b***LEP*, **c***SCD*, and **d***TNF* in tissues of analyzed breeds. Each boxplot shows the first quartile, median, and third quartile, and the whiskers show the minimum and maximum allelic transcript ratio values. s.fat, subcutaneous fat; v.fat, visceral fat; l.m., *longissimus dorsi* muscle; gDNA, genomic DNA; PLW, Polish Large White; PL, Polish Landrace

We first identified animals that were heterozygous for an exonic reporter SNP (rSNP) in each gene, which is necessary to distinguish allelic transcripts and quantify their proportions. Based on the frequencies of heterozygous genotypes in our pig populations (Suppl. Table S2), measurements of allelic ratios were performed in breeds where at least 10 heterozygotes were available, i.e., in PLW, PL, Duroc, and Pietrain for *ACACA*; PLW and PL for *ADIPOR1*, *FASN*, and *TNF*; PLW and Duroc for *LEP* and *SCD*; and PLW, PL, and Duroc for *ME1* (Fig. 1, Suppl. Fig. S1). Pyrosequencing was used as a reliable and sensitive means of studying allele-specific expression (Wang and Elbein 2007). We detected allelic imbalance of *ACACA*, *LEP*, *SCD*, and *TNF* in all breeds and tissues analyzed (Table 1). The most significant differences (*p* < 0.001) were found for *ACACA* in all tissues, for *LEP* in skeletal muscle, and for *TNF* in visceral fat but the effects were breed-specific (Table 1). The bi-directional nature of allelic imbalance of *ACACA*, *LEP*, *SCD*, and *TNF* (Fig. 1) provides evidence that the regulatory elements that affect their allelic expression are not in linkage disequilibrium with exonic rSNPs used to quantify allelic proportions. Mean allelic ratios calculated for each breed and tissue showed that the same allele was overexpressed for *ACACA* (A allele), *LEP* (C allele), and *SCD* (G allele) in all groups tested and for *TNF* (A allele) in PLW breed. Interestingly, this overrepresentation of one allele was also related to its higher frequency in several breeds and the highest effect was observed in case of allelic expression of *ACACA* in Duroc pigs (Suppl. Table S3). This may suggest a synergistic process resulting in preference of this particular allele. For *ADIPOR1*, *FASN*, and *ME1*, there were no significant deviations of allelic transcript levels between cDNA and gDNA in any breed or tissue (data not shown).

**Table 1:** Mean log_10_-transformed allelic ratios ± SD in cDNA derived from subcutaneous fat, visceral fat, and *l. dorsi* muscle, and gDNA for *ACACA*, *LEP*, *SCD*, and *TNF*

Gene	Breed	Subcutaneous fat	Visceral fat	*L. dorsi* muscle	gDNA
*ACACA*	PLW	0.094 ± 0.054***	0.091 ± 0.059**	0.093 ± 0.053***	0.026 ± 0.025
	PL	0.094 ± 0.062***	0.104 ± 0.076***	0.074 ± 0.055**	0.032 ± 0.018
	Duroc	0.101 ± 0.057*	0.122 ± 0.053**	0.094 ± 0.047*	0.039 ± 0.039
	Pietrain	0.113 ± 0.047***	0.118 ± 0.062***	0.101 ± 0.068*	0.041 ± 0.030
*LEP*	PLW	0.085 ± 0.071*	0.076 ± 0.052*	0.105 ± 0.099*	0.036 ± 0.035
	Duroc	0.083 ± 0.048**	0.079 ± 0.078*	0.106 ± 0.057***	0.026 ± 0.021
*SCD*	PLW	0.106 ± 0.044**	0.127 ± 0.109*	0.180 ± 0.116**	0.037 ± 0.032
	Duroc	0.093 ± 0.052**	0.094 ± 0.053**	0.174 ± 0.120**	0.033 ± 0.031
*TNF*	PLW	0.120 ± 0.107**	0.104 ± 0.059***	0.126 ± 0.139*	0.026 ± 0.021
	PL	0.140 ± 0.113**	0.118 ± 0.144*	0.168 ± 0.132**	0.028 ± 0.026

Data were calculated after neutralizing bi-directional character of allelic imbalance. Significant differences between cDNA and gDNA are shown at *p* < 0.05 (*), *p* < 0.01 (**), and *p* < 0.001 (***)

We then focused on searching for epigenetic regulatory elements located in 5′-flanking regions of *ACACA* and *SCD* where CpG islands were annotated according to the reference Sscrofa 11.1 assembly. For analysis of CpG methylation level, we selected samples showing most extreme allelic imbalance (mean log_10_-transformed allelic transcript ratio for *ACACA* in visceral fat: 0.21 ± 0.03; and for *SCD* in visceral fat: 0.23 ± 0.06 and in *l. dorsi*: 0.28 ± 0.05) versus control groups showing similar expression of both alleles (mean log_10_-transformed allelic transcript ratios: 0.02 ± 0.01, 0.04 ± 0.02, and 0.06 ± 0.04, respectively). The study investigated methylation in two CpG islands (CGi) in the *ACACA* promoter region in DNA isolated from visceral fat: CGi1 (five CpG sites) and CGi2 (nine CpG sites). We also investigated a CGi located in the *SCD* promoter region, where six CpG sites were analyzed in visceral fat and *l. dorsi* muscle. The mean percentage of methylated cytosines was low, 3–8% for CGi1 and 6–12% for CGi2 in *ACACA*, and 3–10% for the CGi in *SCD* in visceral fat and *l. dorsi* muscle (Suppl. Fig. S2) which was as expected for actively transcribed genes (Meier and Recillas-Targa 2017). The comparison of mean CpG methylation level between groups revealed no significant effects on allelic imbalance of *ACACA* and *SCD*.

Finally, to study possible association of candidate SNPs in the *TNF* 5′-regulatory region (rs328373700, c.-791C>T) and in 3′UTR of *ACACA* (rs321308225, c.*195C>A) with mRNA expression in two fat deposits and *l. dorsi* muscle, allelic transcript ratios from animals carrying different genotypes were compared. This strategy reduces any confounding effects of *trans*-regulatory and environmental factors on mRNA expression because transcript abundance is compared within the same sample, not between individuals (Forton et al., 2007). The candidate SNPs selected for this analysis were previously reported as associated with fat deposition and carcass traits (Stachowiak et al. 2013; Szydlowski et al. 2011). Due to the genotype distribution in our groups heterozygous for rSNPs (Suppl. Table S4), we could perform this study to test the regulatory effect of rs321308225 (c.*195C>A) SNP in PL breed, only. Although animals carrying CA genotype showed more imbalanced *ACACA* allelic expression than CC homozygotes in all tissues analyzed, the differences were not statistically significant (Suppl. Table S5). We previously reported that *ACACA* shows a distinct expression pattern in subcutaneous fat and *l. dorsi* muscle of PL pigs (Stachowiak et al. 2013), and the positioning of chromosome territory carrying *ACACA* has been correlated with its transcriptional activity in porcine adipocytes (Kociucka et al. 2012) but the mechanism of its tissue-specific regulation remains unknown.

This is the first study that shows differential allelic expression of *ACACA*, *LEP*, *SCD*, and *TNF* in fat deposits and skeletal muscle of postnatal pigs. The recently reported allelic imbalance of two genes, *PPARA* and *PPARGC1A*, out of four analyzed (Stachowiak et al. 2018) suggests that this phenomenon may be widespread among genes involved in regulating lipid metabolism in pigs. Interestingly, *SCD* was previously shown as imbalanced in porcine prenatal skeletal muscle, whereas *LEP* but not *FASN*, *SCD*, and *TNF* displayed differential allelic expression in bovine liver, pituitary, and kidney (Olbromski et al. 2013; Yang et al. 2016). The strategy to search for functional regulatory elements based on analysis of allelic expression has successfully revealed *cis*-regulatory variants governing expression of *IL13* in human lymphoblastoid B cell lines and *ADRB2* in porcine *l. dorsi* muscle (Forton et al. 2007; Murani et al. 2013). Such an approach should also be adopted to investigate the molecular basis of extensive allele-specific expression observed in pig fat tissue (Schachtschneider et al. 2015) or a skeletal muscle.

In conclusion, of seven genes analyzed, *ACACA*, *LEP*, *SCD*, and *TNF*, exhibited significant allelic imbalance in fat deposits and skeletal muscle and these genes are interesting candidates for investigation of *cis*-regulatory factors as potential molecular targets to modulate porcine fatness traits. Such a study should also include other elements than the linear DNA sequence, for example, *cis*-regulatory chromatin modifications or three-dimensional architecture of chromatin domains in the interphase nucleus.

## Electronic supplementary material

ESM 1 (DOC 392 kb)
