# Supplementary material for: Analysis of allele-specific expression of seven candidate genes involved in lipid metabolism in pig skeletal muscle and fat tissues reveals allelic imbalance of ACACA, LEP, SCD, and TNF
Source: J Appl Genet. 2019 Jan 26;60(1):97–101. doi: 10.1007/s13353-019-00485-z (PMC6373405; doi:10.1007/s13353-019-00485-z)
Supplement: Supplementary file 1 — (DOC 392 kb) [file 13353_2019_485_MOESM1_ESM.doc]

**
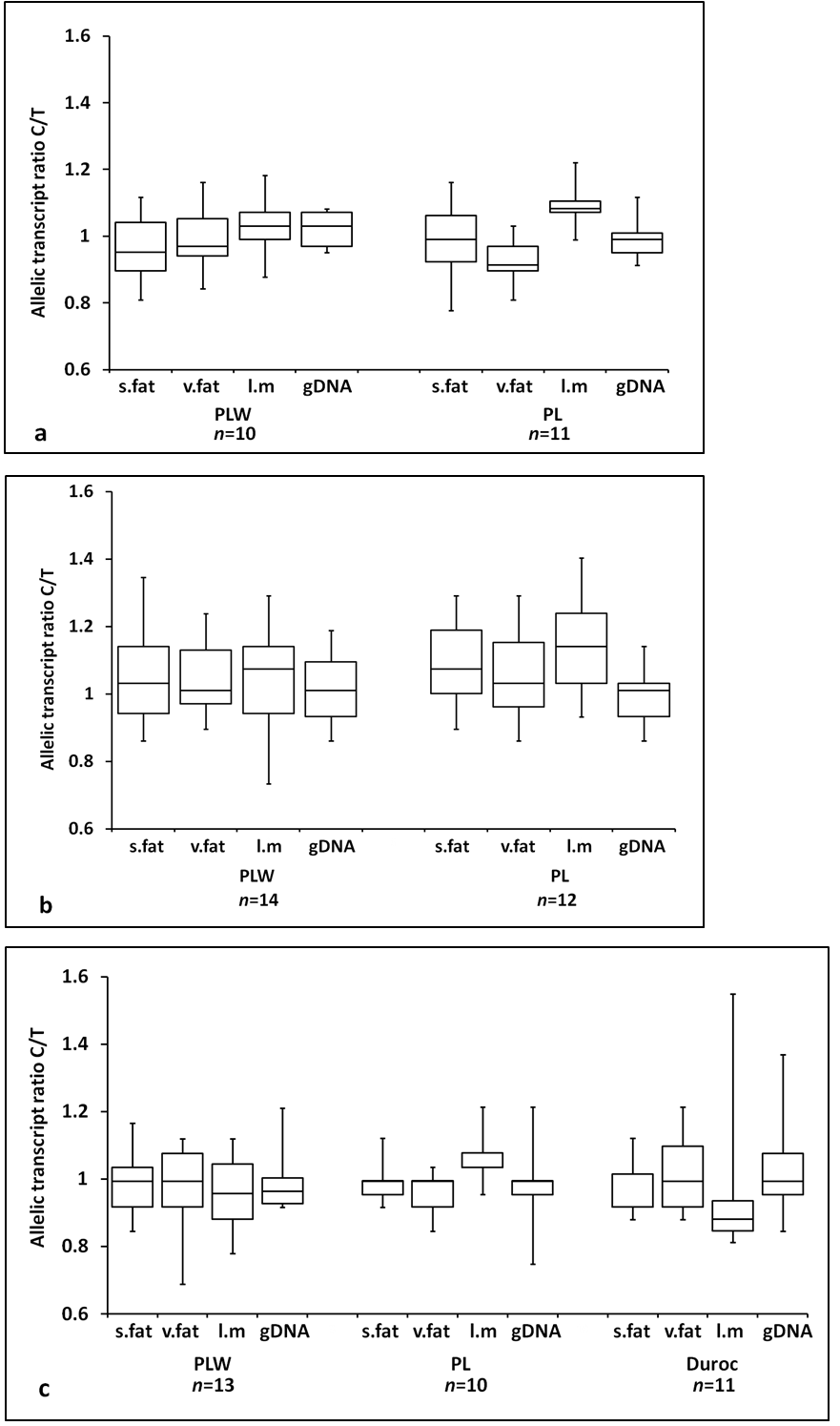
**

**Suppl. Fig. S1** Distribution of allelic ratios for **a**) *ADIPOR1*, **b**) *FASN*, and **c**) *ME1* in tested tissues of analyzed breeds. Each boxplot shows the first quartile, median, third quartile and the whiskers show the minimum and maximum allelic transcript ratio values. s.fat – subcutaneous fat, v.fat – visceral fat, l.m. – *longissimus dorsi* muscle, gDNA – genomic DNA, PLW – Polish Large White, PL – Polish Landrace


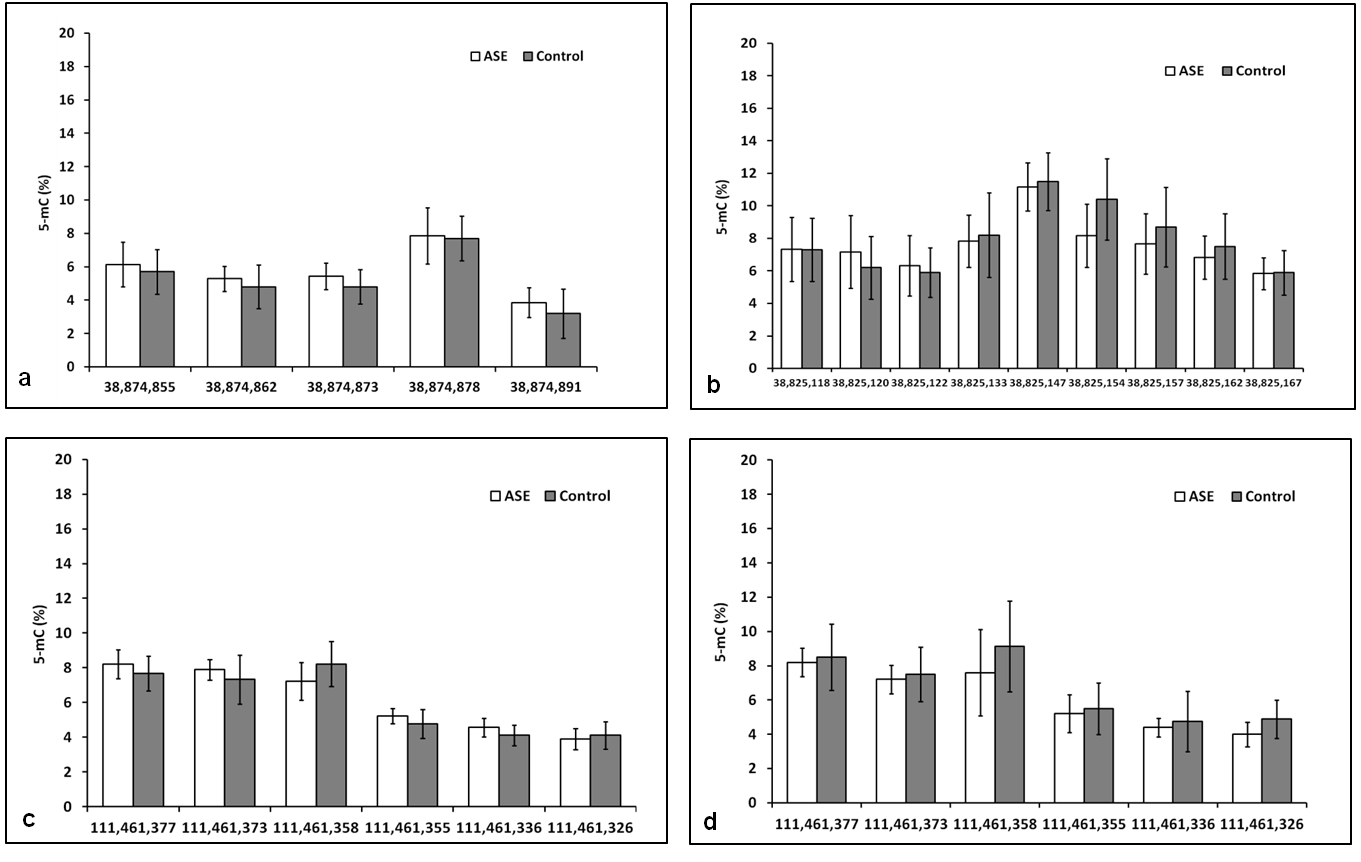


**Suppl. Fig. S2** Mean percentage of 5-methylcytosine (5-mC) ± SD within CpG islands located in 5’-flanking regions of (**a**) *ACACA* (CGi1) in visceral fat; (**b**) *ACACA* (CGi2) in visceral fat; (**c**) *SCD* in *l. dorsi* muscle and (**d**) *SCD* in visceral fat, in samples showing most extreme allele-specific expression (ASE) and control groups with similar expression of both alleles. Genomic positions of particular CpG sites in chromosome 12 for *ACACA* and chromosome 14 for *SCD* are shown according to Sscrofa11.1 reference assembly (NC_010454.4)

**Supplementary Table S1 PCR primers used for genotyping by Sanger sequencing, allele quantification and CpG methylation analysis by pyrosequencing**

| Gene | Type of analysis ^1,2^ | PCR primers (5’→3’) ^3^ | Amplicon length (bp) |
| --- | --- | --- | --- |
| *ACACA (acetyl-CoA carboxylase alpha)* | Genotyping for rSNP rs81303284 (c.*99A>T) and a candidate SNP rs321308225 (c.*195C>A) | F: atggactcgccttccacgtagga  R: tcttaggtcctgggcaagaagagg | 297 |
|  | Allele quantification | F: *ccagtatgttgtgcgtaacctga  R: caactgaagccactgtaatgagac  S: cagcataggaggccc | 104 |
|  | CpG methylation within CGi1 | F: aggggtggtaggaatgttata  R: *aactccaacccctaacct  S: atttttttaggtttttgttgg | 244 |
|  | CpG methylation within CGi2 | F: agaggaatggggtatggt  R: *acctacactaacttaaaaaccaacctatat  S: gaatggggtatggtt | 232 |
| *ADIPOR1(adiponectin receptor 1)* | Genotyping for rSNP rs81508987 (c.759G>A) | F: ctggattattcagggattgcc  R: ctgctcttgctcgccggt | 191 |
|  | Allele quantification | F: cttgtctgccggtgcttagga  R: *ctgggcatttctgccatca  S: caaaccggtcccact | 68 |
| *FASN (fatty acid synthase)* | Genotyping for rSNP rs324640280 (c.339C>T) | F: cggttccttgtgacctgttt  R:ccgtgtctatggtgatgctg | 567 |
|  | Allele quantification | F: cttcgtggcacgagcacc  R: *ctggcagcctatcatgctgta  S: ggtgggtgtgagcag | 110 |
| *LEP (leptin)* | Genotyping for rSNP rs45431504 (c.289T>C) | F: gggtcaccggtttggacttcatcc  R:gcccaggctctccaaggtctcc | 230 |
|  | Allele quantification | F: *tggaaggcagactggtgag  R: ttcatccctgggctccat  S: ccatcctgtcctgagt | 91 |
| *ME1* *(malic enzyme 1)* | Genotyping for rSNP rs328566530 (c.582T>C) | F: ggaactgtgtgccaggattt  R: tctttaaggtatattgctgtgttgc | 261 |
|  | Allele quantification | F: agcatgtggaggggtaaatc  R: *cctcattttctgttccca  S: tgcctgtcatcctgg | 68 |
| *SCD (stearoyl-CoA desaturase)* | Genotyping for rSNP rs334462984 (c.*931C>G) | F: aaaagcacatcccagtgtcc  R: cccccattcttcctcttctc | 475 |
|  | Allele quantification | F: ttagtttgtggcagcagtggtctc  R: *cgggaagatgaaaagagtgaggac  S: aatcggcaactcatg | 161 |
|  | CpG methylation within CGi3 | F: *gtgttttagggttagttttgggttagtat  R: ccctttctcctcctcaactt  S: ctcctcaacttctctt | 157 |
| *TNF (tumor necrosis factor)* | Genotyping for rSNP rs80945725 (c.306A>G) ^3^ | F: tcgagggccaggaggtggaga  R: gacggcgatgcggctgatgg | 280 |
|  | Allele quantification | F: aatgtcaaagccgaggga  R: *tgaagaggacctgggagtagat  S: cgagggacagctcca | 139 |
|  | Genotyping for a candidate SNP rs328373700 (c.-791C>T) | F: ctagcggctggagatacagg  R: agggctcaggtaggggtaga | 490 |
| *ACTB (actin beta)* | Control of gDNA contamination in cDNA samples | F: ggacttcgagcaggagatgg  R: gcaccgtgttggcgtagagg | 329 (gDNA)  234 (cDNA) |

^1^ rSNP=reporter SNP; ^2^ Cgi=CpG island; ^3^ primer sequences from Szydlowski et al. (2011);

^*^ primers labeled at 5’-end with biotin

**Supplementary Table S2** Genotype frequencies of rSNPs in tested pig breeds. For each gene, breeds where further study was possible due to sufficient number of heterozygotes (at least 10) are bold and underlined

|  | Genotype frequency | | |
| --- | --- | --- | --- |
| Breed^1^ | *ACACA,* rs81303284 (c.*99A>T) | | |
|  | AA | **AT** | TT |
| **PLW (n=48)** | 0.33 | **0.29** | 0.38 |
| **PL (n=35)** | 0.23 | **0.57** | 0.20 |
| **Duroc (n=38)** | 0.74 | **0.26** | 0.00 |
| **Pietrain (n=21)** | 0.29 | **0.57** | 0.14 |
|  | *ADIPOR1,* rs81508987 (c.759G>A) | | |
|  | AA | **AG** | GG |
| **PLW (n=48)** | 0.02 | **0.21** | 0.77 |
| **PL (n=35)** | 0.40 | **0.43** | 0.17 |
| Duroc (n=38) | 0.00 | **0.03** | 0.97 |
| Pietrain (n=21) | 0.00 | **0.00** | 1.00 |
|  | *FASN*, rs324640280 (c.339C>T) | | |
|  | CC | **CT** | TT |
| **PLW (n=48)** | 0.23 | **0.44** | 0.33 |
| **PL (n=35)** | 0.43 | **0.40** | 0.17 |
| Duroc (n=38) | 1.00 | **0.00** | 0.00 |
| Pietrain (n=21) | 0.71 | **0.29** | 0.00 |
|  | *LEP*, rs45431504 (c.289T>C) | | |
|  | CC | **CT** | TT |
| **PLW (n=48)** | 0.04 | **0.35** | 0.61 |
| PL (n=35) | 0.00 | **0.14** | 0.86 |
| **Duroc (n=38)** | 0.03 | **0.45** | 0.52 |
| Pietrain (n=21) | 0.00 | **0.19** | 0.81 |
|  | *ME1*, rs328566530 (c.582T>C) | | |
|  | CC | **CT** | TT |
| **PLW (n=48)** | 0.60 | **0.40** | 0.00 |
| **PL (n=35)** | 0.58 | **0.31** | 0.11 |
| **Duroc (n=38)** | 0.43 | **0.39** | 0.18 |
| Pietrain (n=21) | 1.00 | **0.00** | 0.00 |
|  | *SCD*, rs334462984 (c.*931C>G) | | |
|  | CC | **CG** | GG |
| **PLW (n=48)** | 0.04 | **0.23** | 0.73 |
| PL (n=35) | 0.00 | **0.14** | 0.86 |
| **Duroc (n=38)** | 0.42 | **0.40** | 0.18 |
| Pietrain (n=21) | 0.00 | **0.14** | 0.86 |
|  | *TNF*, rs80945725 (c.306A>G) | | |
|  | AA | **AG** | GG |
| **PLW (n=48)** | 0.37 | **0.40** | 0.23 |
| **PL (n=35)** | 0.57 | **0.40** | 0.03 |
| Duroc (n=38) | 1.00 | **0.00** | 0.00 |
| Pietrain (n=21) | 0.90 | **0.05** | 0.05 |

^1^ PLW – Polish Large White, PL – Polish Landrace

**Supplementary Table S3** Mean allelic ratios in fat tissues, skeletal muscle and genomic DNA in heterozygous samples for exonic rSNPs and allele frequencies for rSNPs in all tested populations.

| **Gene (SNP)** | **Breed** | **Mean allelic ratio** | | |  | **Allele frequency in analysed breeds** | |
| --- | --- | --- | --- | --- | --- | --- | --- |
|  |  | **Subcutaneous fat** | **Visceral fat** | ***L. dorsi* muscle** | **GenomicDNA** |  |  |
| *ACACA (*rs81303284, c.*99A>T) |  | **A/T** | | | | **A** | **T** |
|  | PLW | 1.20 | 1.19 | 1.23 | 0.98 | 0.48 | 0.52 |
|  | PL | 1.19 | 1.27 | 1.18 | 0.98 | 0.51 | 0.49 |
|  | Duroc | 1.27 | 1.25 | 1.25 | 1.03 | 0.87 | 0.13 |
|  | Pietrain | 1.29 | 1.25 | 1.23 | 1.05 | 0.58 | 0.42 |
| *LEP* (rs45431504, c.289T>C) |  | **C/T** | | | | **C** | **T** |
|  | PLW | 1.14 | 1.13 | 1.13 | 0.99 | 0.21 | 0.79 |
|  | Duroc | 1.11 | 1.09 | 1.05 | 1.01 | 0.25 | 0.75 |
| *SCD* (rs334462984, c.*931C>G) |  | **C/G** | | | | **C** | **G** |
|  | PLW | 0.90 | 0.77 | 0.68 | 0.96 | 0.15 | 0.85 |
|  | Duroc | 0.92 | 0.89 | 0.76 | 1.03 | 0.62 | 0.38 |
| *TNF* (rs80945725, (c.306A>G) |  | **A/G** | | | | **A** | **G** |
|  | PLW | 1.07 | 1.03 | 1.23 | 1.00 | 0.57 | 0.43 |
|  | PL | 1.07 | 0.96 | 0.92 | 1.00 | 0.77 | 0.23 |

**Supplementary Table S4** Genotype frequencies for candidate SNPs in *ACACA* and *TNF* in heterozygous samples for exonic reporter SNPs (rs81303284 and rs80945725, respectively)

| **Gene** | **Breed^a^** | **Genotype** | |  |
| --- | --- | --- | --- | --- |
|  |  | **CC** | **CA** | **AA** |
| *ACACA*  rs321308225 (c.*195C>A) | PLW (n=14) | 1.00 | 0.00 | 0.00 |
|  | **PL (n=20)** | **0.60** | **0.40** | 0.00 |
|  | Duroc (n=10) | 1.00 | 0.00 | 0.00 |
|  | Pietrain (n=12) | 0.92 | 0.08 | 0.00 |
|  |  | **CC** | **CT** | **TT** |
| *TNF* | PLW (n=15) | 0.07 | 0.93 | 0.00 |
| rs328373700  (c.-791C>T) | PL (n=13) | 0.07 | 0.77 | 0.16 |
|  |  |  |  |  |

^a^ The group used for an association analysis with allelic transcript ratios is marked with a bold font

**Supplementary Table S5** Mean log_10_-transformed allelic ratios for GG (*n*=12) and GT (*n*=8) genotypes for rs321308225 SNP (c.*195C>A) in 3’UTR of *ACACA*. The analysis was performed for PL breed in heterozygous samples for exonic reporter SNP rs81303284 in *ACACA*. Data were calculated after neutralizing bi-directional character of *ACACA* allelic expression

| **Tissue** | **Subcutaneous fat** | | **Visceral fat** | | ***Longissimus dorsi*** | |
| --- | --- | --- | --- | --- | --- | --- |
| **Genotype** | **CC** | **CA** | **CC** | **CA** | **CC** | **CA** |
| **Allelic transcript ratio (log_10_) ± SD** | 0.083  ±0.064 | 0.112  ±0.059 | 0.078  ±0.060 | 0.144  ±0.085 | 0.065  ±0.037 | 0.089  ±0.076 |
| ***p* value** | 0.344 | | 0.084 | | 0.423 | |
